# Supplementary material for: Assessment of activities of daily living in patients post COVID-19: a systematic review
Source: PeerJ. 2021 Apr 6;9:e11026. doi: 10.7717/peerj.11026 (PMC8034364; doi:10.7717/peerj.11026)
Supplement: Supplemental Information 2 [file peerj-09-11026-s002.docx]

**PubMed**

Search date: 10/09/2020; Restricted to studies published from 01/12/2019-10/09/2020

| # | SEARCH TERMS | N° OF RESULTS |
| --- | --- | --- |
| #1 | ((SARS-CoV-2)[Title/Abstract] OR (COVID-19)[Title/Abstract] OR (2019 novel coronavirus infection)[Title/Abstract] OR (COVID19)[Title/Abstract] OR (coronavirus disease 2019)[Title/Abstract] OR (coronavirus disease-19)[Title/Abstract] OR (2019-nCoV disease)[Title/Abstract] OR (2019 novel coronavirus disease)[Title/Abstract] OR (2019-nCoV infection))[Title/Abstract] | 53,180 |
| #2 | (Functional capacity)[Title/Abstract] OR (activities of daily living)[Title/Abstract] OR (Functional independence measure)[Title/Abstract] OR (Barthel index)[Title/Abstract] OR (Lawton Brody)[Title/Abstract] OR (Katz index)[Title/Abstract] OR (ADL)[Title/Abstract] OR (PCFS)[Title/Abstract] OR (Functional scale)[Title/Abstract] OR (Functional status)[Title/Abstract] | 43,087 |
| #3 | ((SARS-CoV-2)[Title/Abstract] OR (COVID-19)[Title/Abstract] OR (2019 novel coronavirus infection)[Title/Abstract] OR (COVID19)[Title/Abstract] OR (coronavirus disease 2019)[Title/Abstract] OR (coronavirus disease-19)[Title/Abstract] OR (2019-nCoV disease)[Title/Abstract] OR (2019 novel coronavirus disease)[Title/Abstract] OR (2019-nCoV infection))[Title/Abstract] AND ((Functional capacity)[Title/Abstract] OR (activities of daily living)[Title/Abstract] OR (Functional independence measure)[Title/Abstract] OR (Barthel index)[Title/Abstract] OR (Lawton Brody)[Title/Abstract] OR (Katz index)[Title/Abstract] OR (ADL)[Title/Abstract] OR (PCFS)[Title/Abstract] OR (Functional scale)[Title/Abstract] OR (Functional status))[Title/Abstract] | 891 |

**((SARS-CoV-2) OR (COVID-19) OR (2019 novel coronavirus infection) OR (COVID19) OR (coronavirus disease 2019) OR (coronavirus disease-19) OR (2019-nCoV disease) OR (2019 novel coronavirus disease) OR (2019-nCoV infection)) AND ((Functional capacity) OR (activities of daily living) OR (Functional independence measure) OR (Barthel index) OR (Lawton Brody) OR (Katz index) OR (ADL) OR (PCFS) OR (Functional scale) OR (Functional status))**

**Embase**

Search date: 05/09/2020; Restricted to studies published from 01/12/2019-05/09/2020

| # | SEARCH TERMS | N° OF RESULTS |
| --- | --- | --- |
| #1 | 'sars cov 2':ab,ti OR 'covid 19':ab,ti OR '2019 novel coronavirus infection':ab,ti OR covid19:ab,ti OR 'coronavirus disease 2019':ab,ti OR 'coronavirus disease-19':ab,ti OR '2019-ncov disease':ab,ti OR '2019 novel coronavirus disease':ab,ti OR '2019-ncov infection':ab,ti | 45,448 |
| #2 | 'functional capacity':ab,ti OR 'activities of daily living':ab,ti OR 'functional independence measure':ab,ti OR 'barthel index':ab,ti OR 'lawton brody':ab,ti OR 'katz index':ab,ti OR adl:ab,ti OR pcfs:ab,ti OR 'functional scale':ab,ti OR 'functional status':ab,ti | 4,812 |
| #3 | (('sars cov 2':ab,ti OR 'covid 19':ab,ti OR '2019 novel coronavirus infection':ab,ti OR covid19:ab,ti OR 'coronavirus disease 2019':ab,ti OR 'coronavirus disease-19':ab,ti OR '2019-ncov disease':ab,ti OR '2019 novel coronavirus disease':ab,ti OR '2019-ncov infection':ab,ti) AND ('functional capacity':ab,ti OR 'activities of daily living':ab,ti OR 'functional independence measure':ab,ti OR 'barthel index':ab,ti OR 'lawton brody':ab,ti OR 'katz index':ab,ti OR adl:ab,ti OR pcfs:ab,ti OR 'functional scale':ab,ti OR 'functional status':ab,ti) | 33 |

**Web of Science**

Search date: 05/09/2020; Restricted to studies published from 01/12/2019-05/09/2020

| # | SEARCH TERMS | N° OF RESULTS |
| --- | --- | --- |
| #1 | ((SARS-CoV-2) OR (COVID-19) OR (2019 novel coronavirus infection) OR (COVID19) OR (coronavirus disease 2019) OR (coronavirus disease-19) OR (2019-nCoV disease) OR (2019 novel coronavirus disease) OR (2019-nCoV infection)) | 32,895 |
| #2 | ((Functional capacity) OR (activities of daily living) OR (Functional independence measure) OR (Barthel index) OR (Lawton Brody) OR (Katz index) OR (ADL) OR (PCFS) OR (Functional scale) OR (Functional status)) | 19,156 |
| #3 | (((SARS-CoV-2) OR (COVID-19) OR (2019 novel coronavirus infection) OR (COVID19) OR (coronavirus disease 2019) OR (coronavirus disease-19) OR (2019-nCoV disease) OR (2019 novel coronavirus disease) OR (2019-nCoV infection)) AND ((Functional capacity) OR (activities of daily living) OR (Functional independence measure) OR (Barthel index) OR (Lawton Brody) OR (Katz index) OR (ADL) OR (PCFS) OR (Functional scale) OR (Functional status)) | 102 |

**Cochrane Library**

Search date: 05/09/2020; Restricted to studies published from 01/12/2019-05/09/2020

| # | SEARCH TERMS | N° OF RESULTS |
| --- | --- | --- |
| #1 | ((SARS-CoV-2) OR (COVID-19) OR (2019 novel coronavirus infection) OR (COVID19) OR (coronavirus disease 2019) OR (coronavirus disease-19) OR (2019-nCoV disease) OR (2019 novel coronavirus disease) OR (2019-nCoV infection)) | 1,348 |
| #2 | (Functional capacity) OR (activities of daily living) OR (Functional independence measure) OR (Barthel index) OR (Lawton Brody) OR (Katz index) OR (ADL) OR (PCFS) OR (Functional scale) OR (Functional status)) | 10,276 |
| #3 | ((SARS-CoV-2) OR (COVID-19) OR (2019 novel coronavirus infection) OR (COVID19) OR (coronavirus disease 2019) OR (coronavirus disease-19) OR (2019-nCoV disease) OR (2019 novel coronavirus disease) OR (2019-nCoV infection)) AND ((Functional capacity) OR (activities of daily living) OR (Functional independence measure) OR (Barthel index) OR (Lawton Brody) OR (Katz index) OR (ADL) OR (PCFS) OR (Functional scale) OR (Functional status)) | 67 |

**CINAHL**

Search date: 05/09/2020; Restricted to studies published from 01/12/2019-05/09/2020

| # | SEARCH TERMS | N° OF RESULTS |
| --- | --- | --- |
| #1 | ((SARS-CoV-2) OR (COVID-19) OR (2019 novel coronavirus infection) OR (COVID19) OR (coronavirus disease 2019) OR (coronavirus disease-19) OR (2019-nCoV disease) OR (2019 novel coronavirus disease) OR (2019-nCoV infection)) | 16,886 |
| #2 | (Functional capacity) OR (activities of daily living) OR (Functional independence measure) OR (Barthel index) OR (Lawton Brody) OR (Katz index) OR (ADL) OR (PCFS) OR (Functional scale) OR (Functional status)) | 3,509 |
| #3 | ((SARS-CoV-2) OR (COVID-19) OR (2019 novel coronavirus infection) OR (COVID19) OR (coronavirus disease 2019) OR (coronavirus disease-19) OR (2019-nCoV disease) OR (2019 novel coronavirus disease) OR (2019-nCoV infection)) AND ((Functional capacity) OR (activities of daily living) OR (Functional independence measure) OR (Barthel index) OR (Lawton Brody) OR (Katz index) OR (ADL) OR (PCFS) OR (Functional scale) OR (Functional status)) | 33 |
