# Supplementary material for: Assessment of activities of daily living in patients post COVID-19: a systematic review
Source: PeerJ. 2021 Apr 6;9:e11026. doi: 10.7717/peerj.11026 (PMC8034364; doi:10.7717/peerj.11026)
Supplement: Supplemental Information 4 [file peerj-09-11026-s004.docx]

**Supplemental file – Rationale and contribution**

1. The rationale for conducting the systematic review

Since COVID-19 began, more than 100 million people have been infected in the world. About 6% of patients develop the disease severely. The first follow-up reports at 3 and 6 months have shown that hospitalization survivors are left with significant physical, cognitive and respiratory sequelae. In recent months, the concept "long COVID" has appeared to group all these persistent sequelae.

In many countries, follow-up and physical rehabilitation programs have been developed in these patients, but we need to have a reliable evaluation to perform the best intervention. Currently, there are no specific tools that assess the activities of daily living (ADL) in these patients (the Post COVID functional status scale recently validated by our group, measures functionality and not ADL). The research works that have appeared have used different tools, in different moments of follow-up and many times they have been scarcely reported since they are used as secondary outcomes, since to date, it is still a priority to avoid the collapse of hospitals and the saturation of health systems.

Given this, the community of professionals who are dedicated to rehabilitation does not know which are the best tools, and for this reason, our research group decided to carry out a systematic review that serves as a starting point for future research work. To date, there is no systematic review that has reviewed the subject.

Therefore, it is necessary to detect the best way to know and understand functional ability during ADL before and after COVID-19. To fill this knowledge gap, in this work, we analyze the most common test reported to date to evaluate the relationship between ADL functional ability and COVID-19. We also describe the factors related to context and intrinsic capacity to provide an overview of the necessity to assess the impact of COVID-19 in ADL limitations. Appropriate assessment scales will help to objectively evaluate the effect of COVID-19 on patients' functional ability over time, leading to optimal care and rehabilitation strategies for affected subjects. At the same time, ADL functional ability status previous to COVID-19 provides a fine-grained understanding of the importance of individual context in the ADL functional status post-COVID-19.

1. The contribution that the meta-analysis makes to knowledge in light of previously published related reports, including other meta-analyses and systematic reviews.

This is the first systematic review on this topic. We reported that all included studies found a reduction of ADL beyond the test or scale used, revealing a vital worsening of functional ability in ADL performance and consequently loss of independence in COVID-19 patients after the acute phase of infection. Functional ability status previous to COVID-19 is crucial for predicting the severity of the disease and mortality. Barthel Index and ADL score were the most used assessment tools across subjects with different intrinsic capacity and context levels.
